# Racemization in cataractous lens from diabetic and aging individuals: analysis of Asp 58 residue in αA-crystallin

**DOI:** 10.18632/aging.203086

**Published:** 2021-06-07

**Authors:** Xiang-Jia Zhu, Ke-Ke Zhang, Wen-Wen He, Jiao Qi, Yi Lu

**Affiliations:** 1 Eye Institute and Department of Ophthalmology, Eye & ENT Hospital, Fudan University, Shanghai 200031, People’s Republic of China; 2 NHC Key Laboratory of Myopia, Key Laboratory of Myopia, Chinese Academy of Medical Sciences, Fudan University, Shanghai 200031, People’s Republic of China; 3 Shanghai Key Laboratory of Visual Impairment and Restoration, Shanghai 200031, People’s Republic of China

**Keywords:** racemization, aspartyl residue, diabetes, aging, cataract, lens, crystallin

## Abstract

Cataract is the leading cause of visual impairment globally. Racemization of lens proteins may contribute to cataract formation in aging individuals. As a special type of age-related cataract (ARC), diabetic cataract (DC) is characterized by the early onset of cortical opacification and finally developed into a mixed type of cortical and nuclear opacification. We compared racemization of Asp 58 residue, a hotspot position in αA-crystallin, from the cortex and nucleus of diabetic and age-matched senile cataractous lenses, by identifying L-Asp/L-isoAsp/D-Asp/D-isoAsp by mass spectrometry. Compared to nondiabetic cataractous lenses, DC lenses showed a significantly increased cortex/nucleus ratio of D-Asp 58, which originated primarily from an increased percentage of D-Asp 58 in the lens cortex of DC. Moreover, patients diagnosed with diabetes for over 10 years showed a lower cortex/nucleus ratio of D-isoAsp 58 in the lens compared with those who had a shorter duration of diabetes, which originated mainly from an increased percentage of D-isoAsp 58 in the lens nucleus of DC with increasing time of hyperglycemia. Further analysis confirmed decreased protein solubility in diabetic cataractous lenses. The different racemization pattern in DC may be distinguished from ARC and influence its phenotype over the protracted duration of diabetes.

## INTRODUCTION

Cataract, characterized by coloration and opacification in the lens, is the leading cause of visual impairment globally. Diabetes mellitus (DM) is a chronic systemic disease that affects nearly one in eight adults worldwide [1]. Because of the increasing population of diabetes, the Asia-Pacific region including China is now considered to have the world’s largest diabetes epidemic [2, 3]. Among various complications related to diabetes, diabetic retinopathy and diabetic cataract (DC) are the main ocular complications that could lead to significant visual impairment. DC has an even greater prevalence and broader influence than diabetic retinopathy [4–6].

Patients with diabetes have an increased incidence of cataracts [1], which mature much earlier compared with the nondiabetic population with age-related cataract (ARC). Cataract surgeons often notice distinct characteristics of DC in clinical practice, including the early onset of opacification in the cortex and high stickiness of lens tissue, leading to increased difficulties in cortex removal during cataract surgery. As a special type of ARC, these unique phenotypes of DC may indicate its different pathogenesis compared with ARC.

Posttranslational modifications (PTMs) of diverse proteins in the lens may be significant causes of cataract [7, 8]. PTMs in proteins separated from elderly human lenses include deamination, phosphorylation, truncation, and racemization [9], of which racemization is the most common [10]. During racemization, the normal L-aspartyl (L-Asp) residues, through a succinimide intermediate, spontaneously convert into other types of isomers (i.e., L-isoAsp, D-Asp, and D-isoAsp) [11]. Then abnormal aggregation, degradation, and partial protein unfolding could be triggered and induced [12].

The liability of racemization in specific sites on lens proteins has been reported previously, including αA-crystallin [13]. In ARC lenses, increased levels of D-amino acids have been identified at Asp residues of αA-crystallin compared with age-matched clear lenses. Of these lenses, Asp 58 in αA-crystallin is the hotspot position [10] [14]. According to our previous study on highly myopic lens, alterations in constituent ratios of four isomers of Asp may be related to the cataract phenotype [15]. Over the past decade, however, most studies on diabetic lens have concentrated on protein glycation. The underlying research on the role of αA-crystallin racemization in DC, which is characterized as earlier onset of cortical opacification and stronger tissue stickiness than ARC, remains unclear.

Moreover, DC usually initiated with the cortical type of opacification, and then gradually progressed into the mixed type of cortical and nuclear opacification. Therefore, we also concentrated on potential effects of the duration of diabetes on the racemization patterns in diabetic lens.

In this study, we first investigated the racemization levels of αA-crystallins in lens tissues obtained from patients with DC to compare to those aging individuals with ARC, in which the duration of diabetes was also considered during comparative analysis. Therefore, to improve our understanding of the cataractogenesis in diabetic and aging lens, we originally reported on the evaluation of variations in racemization at the specific site, Asp 58, of αA-crystallin.

## RESULTS

### Baseline patients’ characteristics

The baseline characteristics of the DC and ARC patients enrolled are summarized in Table 1. We did not observe significant differences between the two groups in terms of age (*P* > 0.05) and axial length (*P* > 0.05). Fasting blood glucose level was 5.6 ± 0.5 mmol/L in the ARC group and 6.2 ± 1.0 mmol/L in the DC group (*P* = 0.143). We graded the lens opacity of each patient according to the Lens Opacity Classification System III (LOCSIII).

**Table 1:** Patient characteristics.

**Parameter**	**Age-related cataract**	**Diabetic cataract**	***P*-value**
**Patients (*n*)**	10	10	
Mean age (y) ± SD^*^	74.3 ± 4.0	73.0 ± 5.8	> 0.05
**Gender (male/female)**	5/5	5/5	> 0.05
**Axial length (mm)**	22.93 ± 0.43	22.86 ± 0.55	> 0.05
**Serum glucose level (mmol/L)**	5.6 ± 0.5	6.2 ± 1.0	> 0.05
LOCSIII^**^	2.80 ± 0.63 (C) 1.60 ± 1.51 (N)	3.10 ± 0.74 (C) 2.10 ± 1.60 (N)	> 0.05 > 0.05

Abbreviation: ^*^SD: standard deviation.

Abbreviations: ^**^LOCSIII: Lens Opacity Classification System III; C: cortical grading; N: nuclear grading.

### Quantification of Asp 58 racemization in the lens of diabetic and aging individuals

**Figure 1:**
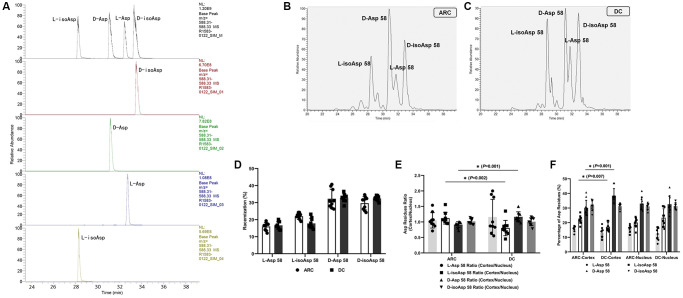
(**A**) Representative LC-MS/MS trace showing the separation of the four Asp isomers of the αA-crystallin tryptic peptide (55–65) TVLDSGISEVR. Peptides containing D-Asp, D-isoAsp, L-Asp, or L-isoAsp at position 58 were synthesized. To measure racemization in αA-crystallin, all forms of the peptide were summed and modifications for each were expressed as a% of the total peak area. (**B**) Representative graphs showing the separation of the four Asp 58 isomers in αA-crystallin of ARC lenses. (**C**) Representative graphs showing the separation of the four Asp 58 isomers in αA-crystallin of DC lenses. (**D**) The percentage of each Asp 58 isomer in αA-crystallin from lenses of patients with ARC and DC. (**E**) The cortex/nucleus ratio of each Asp 58 isomer in αA-crystallin from cortex and nucleus of ARC and DC lenses after dissection. (**F**) The percentage of each Asp 58 isomer in αA-crystallin from cortex and nucleus of ARC and DC lenses after dissection.

In Figure 1A, LC-MS/MS analysis of the four peptide standards shows that the intensities of some fragment ions differed between the Asp and isoAsp isomers. As each peak was confirmed to be the Asp 58 tryptic peptide, we spiked a tryptic digest with synthetic peptide standards to ensure that the elution order had not been altered. After three rounds of validation using peptide standards mixture and each simple component, we sequentially identified the four peaks as L-isoAsp 58 containing peptide, D-Asp58 containing peptide, L-Asp 58 containing peptide, and D-isoAsp 58 containing peptide (Figure 1A). LC-MS/MS spectra of the peaks from the human lens digests of patients with ARC and DC (Figure 1B and 1C) revealed the same MS/MS spectra as those of the corresponding standards, thus confirming their identification. The MS/MS spectra of Asp isoforms containing peptide from ARC and DC patients were also provided (see Supplementary Figure 1 and Supplementary Figure 2).

Using half of each lens tissue (without separating the cortex and nucleus) in each group, we calculated the average racemization rates for each Asp 58 isomer (Figure 1D). The average D/L-isomers ratio in the DC group was 1.98 ± 0.30, and the ratio in the ARC group was 1.67 ± 0.35 (*P* = 0.048). We did not find any significant differences in αA-crystallin of lenses between the two groups concerning the levels of the four different Asp 58 isomers (all *P* > 0.05).

Regarding the residual half of each lens tissue, we dissected this tissue into two regions, outer (cortex) and inner (nucleus), using the trephine (as described in the method section) and separately analyzed the two parts. We compared the percentage and the cortex/nucleus ratio of the Asp 58 residue levels in the two groups (Figure 1E and 1F). Note that the cortex/nucleus ratio of L-isoAsp 58 was lower in the DC group than in the ARC group (0.77 ± 0.24 and 1.12 ± 0.18, respectively, *P* = 0.002), which originated mainly from the decreased percentage of L-isoAsp 58 in the cortex of DC. In contrast, the cortex/nucleus ratio of D-Asp 58 was higher in the DC group than in the ARC group (1.21 ± 0.20 and 0.91 ± 0.09, respectively, *P* = 0.001), which originated mainly from the increased percentage of D-Asp 58 in the cortex of DC. In the lens cortex of DC, D-Asp 58 accounted for the main proportion (as high as 38.47% in average) of the four Asp isomers in αA-crystallin. The percentage was significantly lower (30.15% in average), however, in the lens cortex of ARC. D-Asp was considered as the trigger of abnormal aggregation, degradation, and partial protein unfolding during aging. Thus, we inferred that in the lenses of patients with DC, the elevated conversion from L-isomers to D-Asp 58 in the racemization process in the lens cortex accounted for the primary difference in racemization pattern of αA-crystallin, compared with the lenses of patients with ARC.

**Figure 2:**
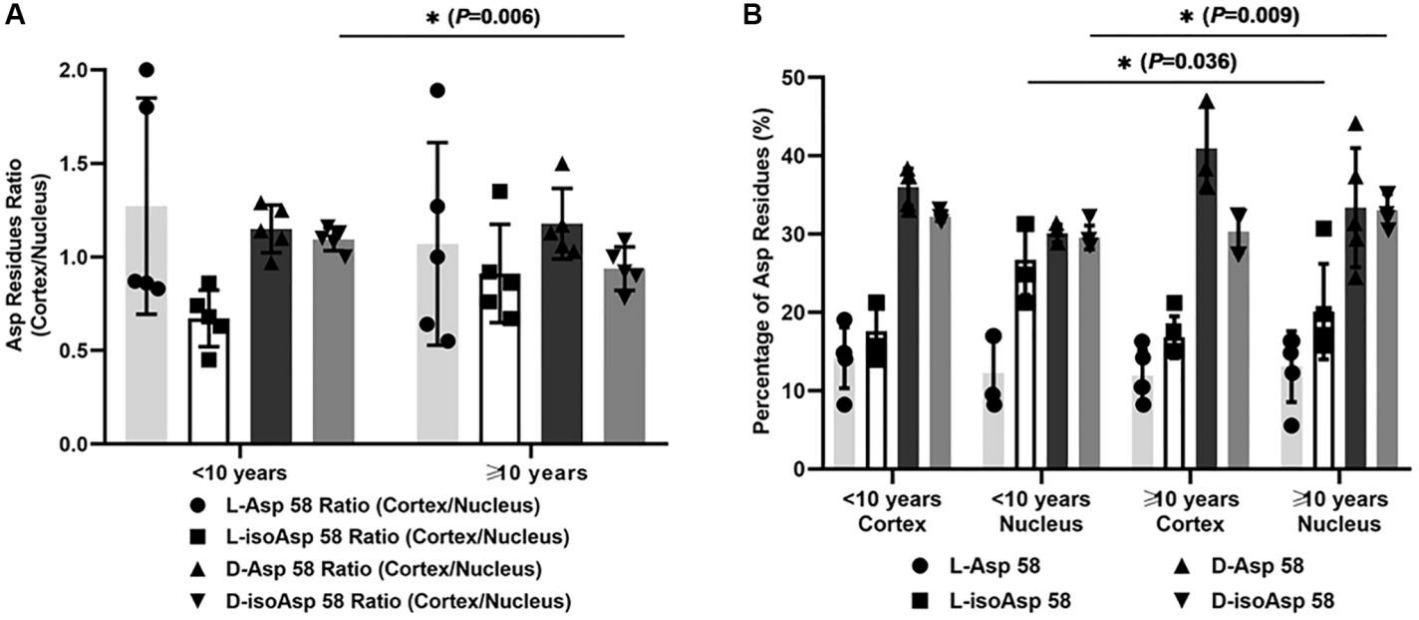
(**A**) The cortex/nucleus ratio of each Asp 58 isomer in αA-crystallin of two subgroups of DC lenses according to the duration of diabetes (less than ten years and more than ten years). (**B**) The percentage of each Asp 58 isomer in αA-crystallin of two subgroups of DC lenses according to the duration of diabetes (less than ten years and more than ten years).

Moreover, we analyzed the correlation between the duration of diabetes and the ratio of different Asp 58 isomers to evaluate the possible influence of longer period of hyperglycemia status on racemization (Figure 2A and 2B). When we divided the patients with DC into two subgroups according to the duration of diabetes (less than ten years and more than ten years), we found that the cortex/nucleus ratio of D-isoAsp 58 was significantly lower in the subgroup with a duration of disease less than ten years (*P* = 0.006, Figure 2A), which originated mainly from the increased percentage of D-isoAsp 58 in the nucleus of DC (from 29.57% ± 1.52% to 33.08% ± 2.02%) with the increased duration of hyperglycemia (Figure 2B). We did not find a significant difference in the comparisons of the cortex/nucleus ratio of other Asp isomers between the two subgroups (L-Asp 58, *P* = 0.542; L-isoAsp, *P* = 0.214; D-Asp 58, *P* = 0.417). According to the percentage of four Asp 58 isomers, when the hyperglycemia status persisted for a longer time, the elevated conversion of L-isomers to D-isoAsp 58 in lens nucleus of DC patients was the primary reason for this difference in the racemization pattern of αA-crystallin.

### Solubility changes in the lens of diabetic and aging individuals

**Figure 3:**
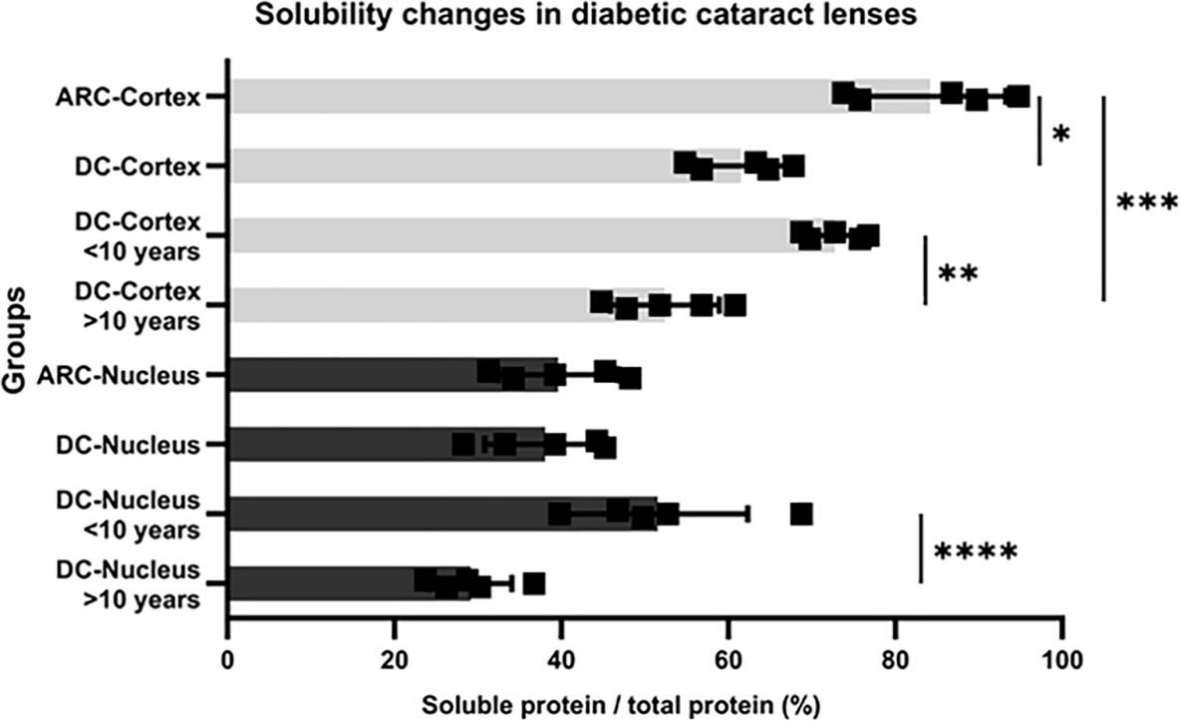
Protein solubility changes in lenses of ARC and DC patients (^*^P = 0.005, ARC cortex vs. DC cortex; ^**^P = 0.013, DC cortex < 10 years vs. DC cortex > 10 years; ^***^P < 0.001, ARC cortex vs, DC cortex > 10 years; ^****^P = 0.007, DC nucleus < 10 years vs. DC nucleus > 10 years).

We measured the total amount of soluble and insoluble protein in a subset of the treated human lenses by BCA assays, as shown in Figure 3. In the cortex of DC lenses over ten years, the average ratio of soluble protein-to-total protein significantly decreased to 52.80%, compared with those in the cortex of ARC lenses (84.76%) and of DC lenses less than ten years (71.78%) (*P* < 0.001, ARC cortex vs. DC cortex > 10 years; *P* = 0.013, DC cortex < 10 years vs. DC cortex > 10 years). Moreover, in the nucleus of DC lenses over ten years, the ratio of soluble protein-to-total protein significantly decreased to 28.74%, compared with those in the nucleus of DC lenses less than ten years (49.74%) (*P* = 0.007). We did not observe a significant difference in the soluble and total protein ratio between the DC lenses less than ten years and the nucleus of the ARC lenses.

## DISCUSSION

The number of adult patients with diabetes has quadrupled in less than four decades. From 108 million in 1980, the number of diabetic cases worldwide skyrocketed to 422 million in 2014 [16]. Cataract is the most common complication of diabetes, leading to visual impairment in people with diabetes worldwide [17]. Patients with diabetes underwent cataract surgery with a high corresponding rate of 12.4 cases per 1000 person-years [18]. As a special type of ARC, DC is characterized by an earlier onset of opacification and higher stickiness in the lens cortex. When the hyperglycemia status persisted, the mixed type of cortical and nucleus opacification would gradually become dominant. Therefore, we explored the possible intrinsic mechanism leading to the occurrence of different phenotypes in DC.

Posttranslational modifications in various lens proteins may be an important cause of DC [7, 8]. Proper protein synthesis and the maintenance of equilibrium is required by the unaltered transparency of lens between molecular compounds, water content, and other constituents of the lens. Chemical agents and factors disturbing the equilibrium induce cataract, especially metabolic disorders, including diabetes. Over the past decade, previous studies have concentrated on protein glycation in diabetic lenses. Elevated blood sugar levels in diabetes are correlated with the accumulation of cytotoxic advanced-glycation end products (AGEs). Hyperglycemia, along with limited cell proliferation and an oxidative environment in many ocular tissues, encourages formation and precludes dilution of AGEs and associated damage by cell division [19]. These circumstances make some ocular tissues vulnerable to glycation-derived damage, and eventually, they may result in cataract formation and development. Accumulating evidence in recent years, however, has shown that racemization, which is the most abundant PTM in the lens, has an inestimable influence on lens protein denaturation, which may accelerate the onset and development of DC. In patients with diabetes, the nonenzymatic reactions, also termed the Maillard reaction, alter the biological and chemical properties of biomolecules [20]. Some studies have revealed that imidazolidinones obtained from glucose and small peptides were almost completely protected from the action of enzymes in serum, with the predominant route of degradation being spontaneous hydrolysis to initial sugar and a peptide compound [20]. Meanwhile, high glucose levels have been found to increase the racemization of Asp residues by increasing the apparent rate of interconversion of the L-and D-enantiomers [21]. However, it is unlikely to be a reaction of glucose with an amino group as in the free amino acids, because the alpha amino group will not be available in the protein. One possibility is that diabetes might change the hydration of the lens due to sorbitol accumulation and in this way alter the packing of the crystallins. Many reports in the literature suggest that the hydrated state of the lens is linked to cataract and recently direct evidence has emerged linking lens swelling to cataract [22]. Cataract formation in diabetic lenses has been attributed to polyol-osmotic pressure-generated influx of water [23]. We speculated that if the lens hydration state and the environment around the protein is altered, it could change the spontaneous chemistry that produces the Asp isomers. It is noted that in young human lenses under teenage years the rate of racemization is significantly faster than in adult lenses [10]. If the lens is stressed more, as in diabetes, it might cause more chaperone binding of α-crystallin to denatured proteins and this could affect the spontaneous chemistry that leads to Asp isomerization, which could also be a possible hypothesis on the alteration of spontaneous chemical reaction in diabetes. Although we still do not fully understand the processes responsible for the pattern of isomers obtained from lens protein digestion, we speculated that racemization may play an unneglectable role in the pathogenesis of DC.

Racemization of amino acids in long-lived proteins takes place in various human tissues [24]. Protein misfolding, aggregation, and insolubilization could lead to many age-related diseases [25, 26], including cataract. The intrinsic instability of some amino acid residues is considered to be a key factor contributing to protein denaturation in aging organisms. As the most well-known tissue with long-lived proteins, the lens is considered to be an optimal model to investigate protein PTMs with a high concentration of crystallins and decreased protein turnover [27]. As indicated previously, Asp residues particularly may be “hotspots” in unstructured regions of proteins, which therefore are susceptible to modification over time, including racemization. In addition, Asp racemization in αA-crystallin of highly myopic cataract lenses also has been studied [14, 15, 24, 28]. The specific conversion patterns in Asp isomers in DC, a unique cataract phenotype, remain unknown.

**Figure 4:**
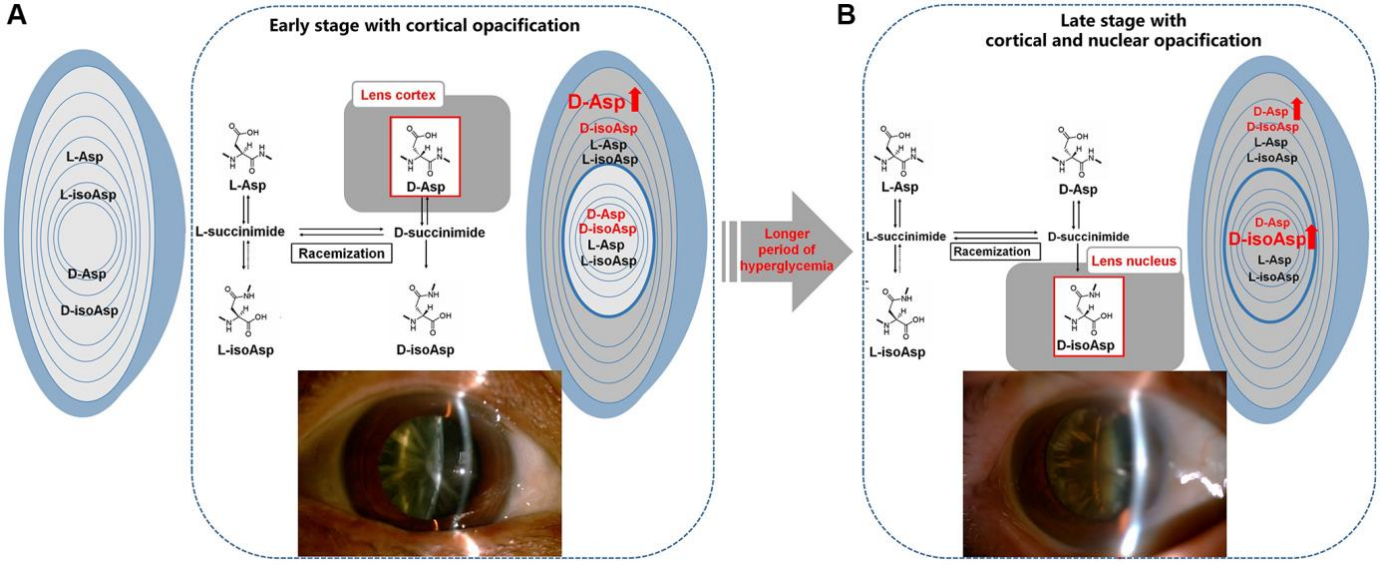
**Illustration of the normal L-Asp 58 spontaneously converted to L-isoAsp 58, D-Asp 58, and D-isoAsp 58 in diabetic lenses.** (**A**) The main difference in racemization at Asp residues in the lenses of DC patients was an increased percentage of D-Asp 58 in the cortex, leading to the early stage of cortical opacification in DC. (**B**) Over the protracted duration of diabetes, the percentage of D-isoAsp 58 in the nucleus increased, leading to the further nuclear opacification on the original basis of cortical opacification in the late stage of DC. L-Asp = L-Asp 58, L-isoAsp = L-isoAsp 58, D-Asp = D-Asp 58, D-isoAsp = D-isoAsp 58.

In our study, we found no significant difference in the percentage of each Asp 58 isomer in the whole lens between the DC and ARC groups. When we divided human lens tissues into the cortical and nuclear regions, different conversion patterns of Asp 58 isomers were revealed. Here, we first demonstrated that the cortex/nucleus ratio of D-Asp 58 was higher in DC than in ARC, which originated mainly from the increased percentage of D-Asp 58 in the lens cortex of DC patients. Moreover, compared with ARC, patients with DC clinically present with cortical cataract in an earlier stage, which is characterized by a more severe opacification and stickiness in the property of the lens cortex. Therefore, the overloaded accumulation of D-Asp 58 in the cortex may trigger the earlier onset of cortical opacification in patients with diabetes (Figure 4A).

Gradual alterations in racemization patterns may also influence the DC phenotypes over the protracted duration of diabetes. The patients who were diagnosed with diabetes for more than ten years showed increased accumulation of D-isoAsp 58 in the lens nucleus compared with those who were diagnosed for less than ten years. Several seemingly independent biochemical pathways overactivated during persistent hyperglycemia in diabetes may have originated from one common abnormality caused by intracellular excess glucose flux (i.e., mitochondrial overproduction of reactive oxygen species, ROS) [29, 30]. Thus, the PTMs of the lens proteins may progress much more rapidly in DC than in ARC because of the persistent damage from ROS. These PTMs, including racemization, have the potential to disrupt the structural and functional properties of crystallins and to contribute to the formation of lens opacification [31]. On the basis of original cortical opacification, the gradually overloaded accumulation of D-isoAsp 58 in the nucleus may play a potential role in the late stage of DC with cortical and nuclear opacification (Figure 4B).

In the case of Asp, four distinct isomeric forms, D-Asp, D-isoAsp, L-isoAsp, and L-Asp, could be spontaneously produced at each site [28]. These mutual conversions likely affected the secondary and tertiary structure, and the quaternary arrangement as well, which in turn affected properties, including solubility and interaction with other proteins [12, 13]. The presence of the D-isomers triggered the partial protein unfolding and abnormal aggregation with decreased protein solubility in lens, contributing to a disease state. Previous studies, including the studies of Fujii et al. and Hooi et al., have intensively analyzed the racemization of Asp 58 in lenses of normal and ARC patients, and D-isoAsp 58 was the most dominant D-isomer [14, 32, 33]. According to our study, based on the protein solubility data, the increased percentage of D-Asp 58 in the cortex mainly contributed to the cortical opacification in the early stage of DC, whereas the elevated percentage of D-isoAsp 58 in the nucleus may have contributed to the progression of nuclear opacification in the late stage of DC. Therefore, our study provided a possible explanation for the effect of Asp 58 residue on different phenotypes of DC.

In conclusion, compared to normally aging individuals, we detected an increased percentage of D-Asp 58 isomer in αA-crystallin in the cataractous lens cortex of diabetic patients. We also found an increased percentage of D-isoAsp 58 isomer in the cataractous lens nucleus of patients who had a longer duration of diabetes. Therefore, the different racemization pattern in DC may be distinguished from ARC and also may have influenced its phenotype over the protracted duration of diabetes.

## METHODS

### Ethics statement

Our investigation was conducted in accordance with the Declaration of Helsinki and according to national and international guidelines. It was approved by the authors' institutional review board. All of the subjects signed an informed consent.

### Sample collection

Between March 1, 2016, and December 31, 2017, we collected ten lenses from patients with DC and ten lenses from age-matched patients with ARC from small-incision cataract extraction surgery in Eye and ENT Hospital, Fudan University, Shanghai, China, and Jinjiang Guangming Hospital, Jiangsu, China. Patients with axial lengths from 22.0 to 24.5 mm were included.

The ARC patients who served as controls were required not have been diagnosed with systemic diseases such as diabetes. Patients with a history of ocular surgery or trauma and those who had glaucoma or uveitis were excluded, as were those with rupture of the posterior capsule during cataract surgery.

The DC patients should meet the following inclusion and exclusion criteria. We defined the DM according to the American Diabetes Association 2010 criteria, including fasting plasma glucose level greater than 126 mg/dL (6.3 mmol/L), 2-hr plasma glucose level greater than 200 mg/dL (after 75 g glucose), or HbA1c level greater than 6.5%. All of the patients with DC had received clear guidance on glucose control and regular medication of melbinum since their diagnosis. To ensure that all of the enrolled patients with DC shared effective glucose control and to avoid severe complications related to diabetes after cataract surgery, we enrolled only those patients on a regular melbin medication regimen and who had a fasting blood glucose level below 149 mg/dL (8.3 mmol/L). The patients with diabetes were separated into two subgroups according to the duration of diabetes, in which five of the patients had diabetes for less than 10 years and the other five for more than 10 years. The other exclusion criteria included uveitis, glaucoma, previous surgical history in eyes, and systemic diseases despite diabetes.

Before surgery, we graded the cortical and nuclear opacity according to the LOCSIII cataract grading system. All the ophthalmic examinations were accomplished thoroughly. We separated each lens into two halves in average. For one half of the lens, we integrated and used the nuclear and cortical region for analysis. For the other half of the lens, we used a pre-cooled 4.5-mm-diameter trephine coring through the visual axis (see Supplementary Figure 3). The separated cortex and nucleus of the human lenses were individually assessed in the following analysis.

### Protein extraction and digestion

We homogenized samples using 150 mM Tris-HCl, 4%SDS, 0.1% v/v protease inhibitor mixture (Merck), and 1 mM dithiothreitol (DTT) using a Dounce homogenizer, and then the samples were sonicated on ice. We evaluated the protein contents by bicinchoninic acid (BCA) protein assay reagent (Beyotime, Wuhan, China) after they were clarified by centrifugation at 16000 × g at 25°C for 10 min. We stored the supernatants at –80°C until use. Proteins were digested using the filter-aided sample preparation procedure. Then proteins (200 mg) were diluted in 200 mL uric acid (UA) buffer (8 M Urea, 150 mM Tris-HCl, pH 8.0) and transferred onto a 10-kDa ultrafiltration filter. After 15-min centrifugation at 14,000 g and washing with 200 mL of UA buffer, we removed DTT and other low-molecular-weight components. The samples were incubated in the dark for 30 min with 50 mM iodoacetamide in UA buffer (100 μL) before centrifugation at 14,000 × g for another 10 min. We washed the filters with 100 μL UA buffer three times and then with 100 μL dissolution buffer twice. Finally, we collected the resulting peptides as a filtrate after adding 2 μg trypsin (Promega, Madison, WI, USA) for digestion overnight at 37°C.

### Peptides synthesis and identification

The four isomers of aspartate in TVLDSGISEVR corresponded to tryptic peptide 55–65 of human αA-crystallin with L-isoAsp/D-Asp/D-isoAsp at position 58 [14] and were purchased from GL Biochem (Shanghai, China). Using a C18 column (GS-120-10-C18-AP, 30 × 250 mm, DiKMA, Beijing, China), we synthesized the peptides (solvent A: 0.1% Trifluoroacetic in 100% acetonitrile, solvent B: 0.1% Trifluoroacetic in 100% water; flow rate: 25.0 mL/min; wavelength: 220 nm; volume: 10 mL). With a flow rate of 1 mL/min, we applied analytical high-performance liquid chromatography (HPLC) using a C18 column (Kromasil, 4.6 × 250 mm, DiKMA, Beijing, China) for peptide purification. We then identified the peptides by mass spectrometry (LCMS-2000, Shimadzu, Kyoto, Japan).

### Racemization analysis of αA-crystallin

Coupled to an Easy nLC (Thermo Fisher Scientific), we performed mass spectroscopy experiments using a LTQ OrbitrapVelos Pro mass spectrometer. Each fraction (2 μL) extracted from the lenses of patients with ARC and DC was loaded onto a reverse phase trap column (75 μm × 2 cm; 5 μm; 100 Å; C18, Thermo Scientific, Bremen, Germany) with buffer A (0.1% formic acid) before elution from the analytic column (75 μm × 15 cm; 3 μm; 100Å; C18, Thermo Scientific) with the following gradient buffer B (0.1% formic acid with 80% acetonitrile) at a flow rate of 3 uL/min. The gradient was 41 min in 0%–20% buffer B, 51 min in 20%–40% buffer B, 53 min in 100% buffer B, and finally maintained in 100% buffer B for 60 min. The HPLC eluates were directly electrosprayed into the mass spectrometer (MS).

We calibrated the mass spectrometer before analysis using standard compounds and operated in the data-dependent mode. We acquired the MS spectra in the m/z range of 160 to 1350 and acquired survey scans in an Orbitrap mass analyzer at a mass resolution of 60,000 at 400 m/z. We selected targeted peptides mass list of 588.32 acquired in the survey scans for CID fragmentation with normalized collision energy of 27%, resolution for CID MS/MS spectra was set to 15,000 at m/z 400, spray voltage was set to 1.8 Kv, and ion transfer tube temperature was set to 250°C.

We calculated four peptide forms and presented modifications for each (L-Asp, L-isoAsp, D-Asp, and D-isoAsp) as the percentage of the total peak area. We determined the L- to D-form ratio on the basis of the peak area in each graph. The ratio of D/L-isomers as (D-Asp + D-isoAsp)/(L-Asp + L-isoAsp) was calculated for further statistical analysis in cataractous lens from diabetic and aging individuals.

### Measurement of solubility changes in human lenses

We homogenized the lens tissues in 50 μL of buffer A (10 mM phosphate, pH 7.0 with 1:1000 protease inhibitor and 0.1 mM ethylene glycol tetraacetic acid). We centrifuged the homogenate at 100,000 g for 30 min at 4°C and collected the supernatant and homogenized the pellet with buffer A another three times, to yield a total volume of approximately 200 μL containing the water-soluble protein. The insoluble pellet was then suspended in 50 μL of buffer B (10 mM Tris, pH 8.0 with 8M urea), homogenized and centrifuged at 20,000 g for 30 min at 4°C. We re-extracted the pellet with 50 μL of buffer B and combined the supernatants to yield the water-insoluble fractions. To evaluate the protein content of the water-soluble and water-insoluble fractions, we measured the protein content in the supernatant and total homogenate in triplicate by BCA assay (Pierce, Rockford, IL, USA). We then calculated the ratio of soluble versus total protein to determine the solubility changes in the cortex and nucleus of human lenses.

### Statistical analysis

Data were shown as mean values ± standard deviations (SD). We performed statistical analyses using SPSS software (v20.0, SPSS Inc., Chicago, IL, USA) and calculated peak areas of specific peptides by a mean-smoothing method. For αA-crystallin, we calculated all forms of the peptide (L-Asp 58, L-isoAsp 58, D-Asp 58, and D-isoAsp 58) and presented modifications for each as the percentage of the total peak area. We analyzed the difference between the ARC and DC groups using independent-samples *t*-test. A *P*-value of < 0.05 was considered statistically significant in all cases.

## Supplementary Materials

Supplementary Figures
