# Supplementary material for: Racemization in cataractous lens from diabetic and aging individuals: analysis of Asp 58 residue in αA-crystallin
Source: Aging (Albany NY). 2021 Jun 7;13(11):15255–68. doi: 10.18632/aging.203086 (PMC8221327; doi:10.18632/aging.203086)
Supplement: Supplementary Figures [file aging-13-203086-s001.pdf]

SUPPLEMENTARY FIGURES

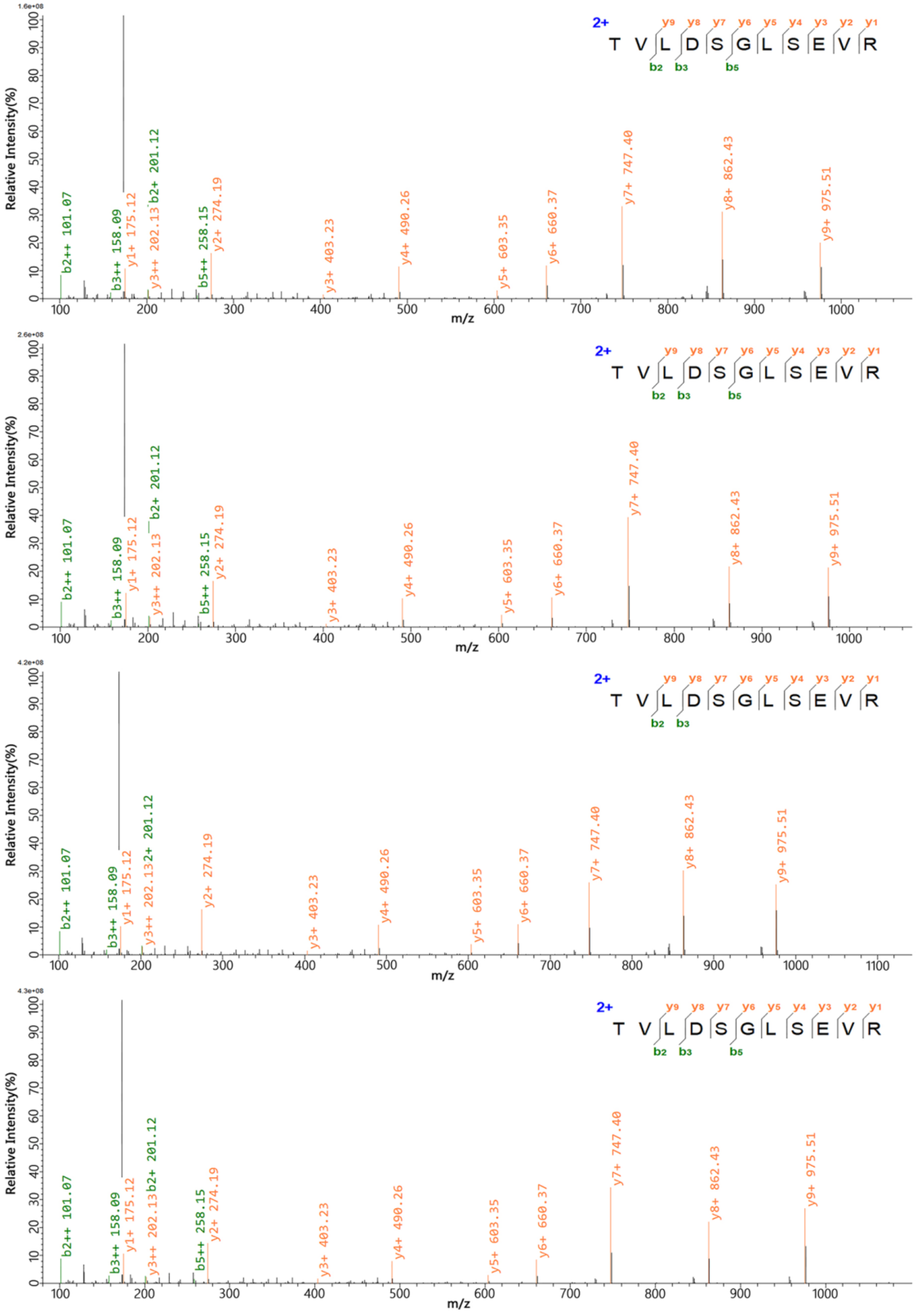

Supplementary Figure 1. The MS/MS spectra of Asp isoforms containing peptide from ARC patients.

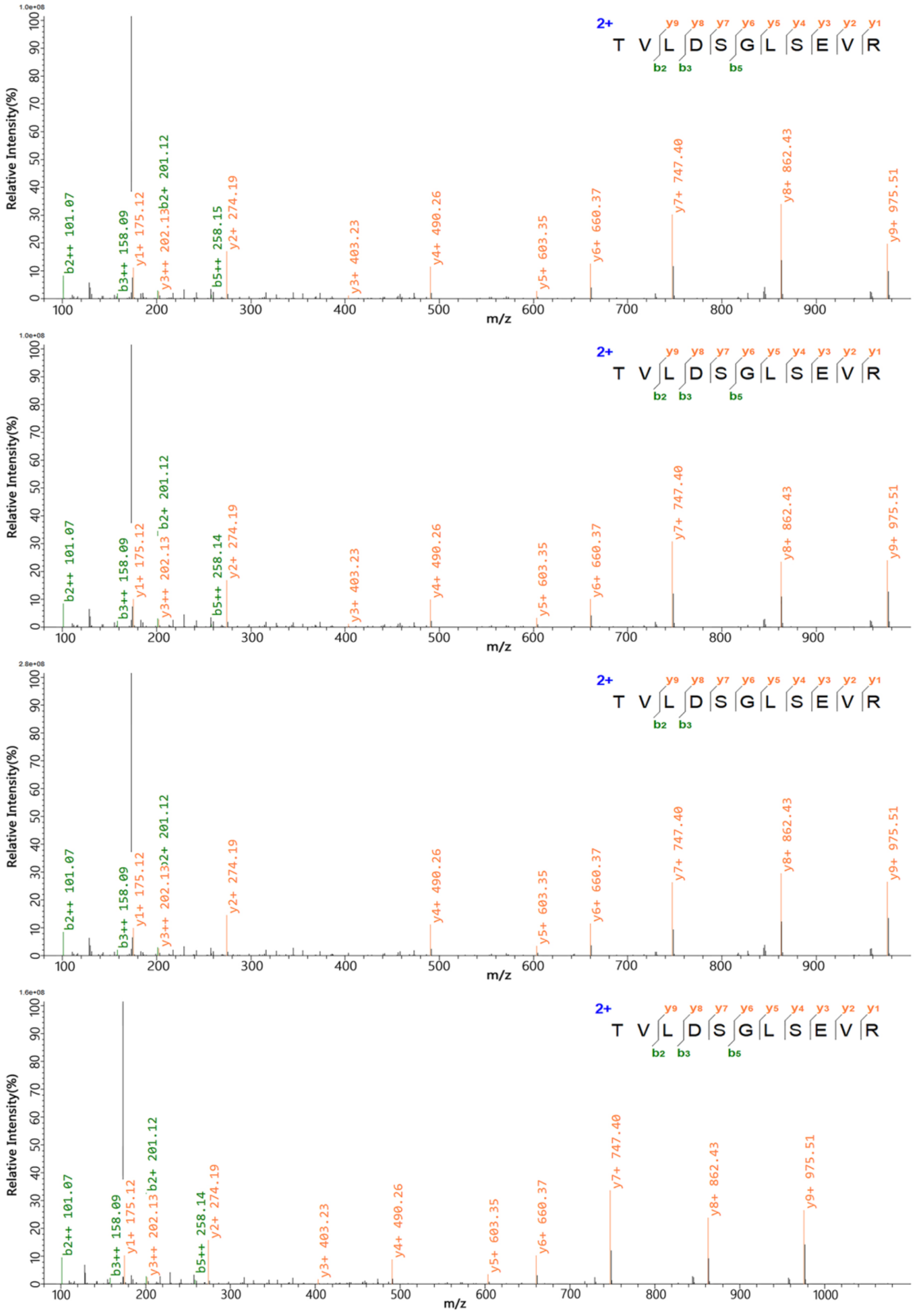

Supplementary Figure 2. The MS/MS spectra of Asp isoforms containing peptide from DC patients.

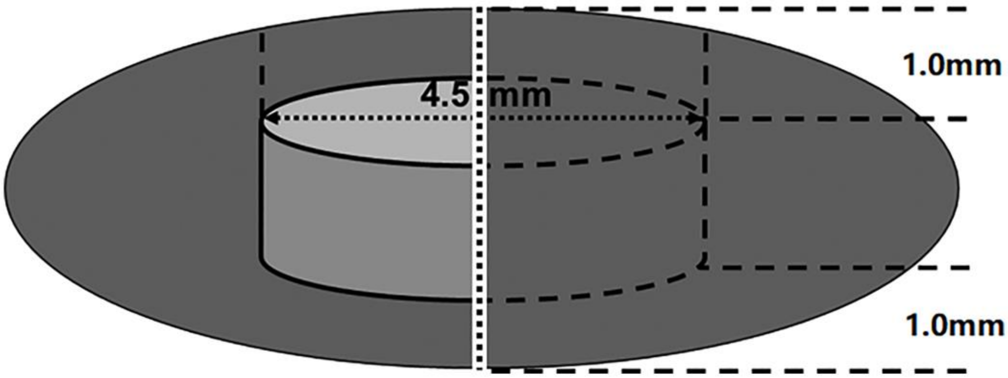

Supplementary Figure 3. Dissection of human lenses.
